# Development of a Cricothyrotomy Virtual Reality Training Module

**DOI:** 10.7759/cureus.90955

**Published:** 2025-08-25

**Authors:** Allyson Molzahn, Tara Burke-Alexander, David Biffar, Allan Hamilton, Kate E Hughes

**Affiliations:** 1 Health Sciences, University of Arizona, Tucson, USA; 2 Surgery, University of Arizona, Tucson, USA; 3 Emergency Medicine, University of Arizona, Tucson, USA

**Keywords:** cricothyrotomy, emergency medicine medical education, simulation in medical education, teaching in emergency medicine, virtual reality simulation

## Abstract

Introduction

Cricothyrotomy is a rare but critical emergency procedure, making simulation-based training essential. Traditional methods, such as cadavers and synthetic models, provide hands-on experience but are limited by time and instructor availability. Virtual reality (VR) offers an immersive, low-risk environment for repeated practice, which is particularly valuable for high-acuity, low-frequency procedures. This study developed and evaluated a VR cricothyrotomy simulation to assess its educational value, realism, and usability.

Methods

The VR cricothyrotomy simulation and headset were provided by 8Chili, Inc. (Milpitas, CA, USA). The module was developed with input from a multidisciplinary team of healthcare professionals. Fifteen emergency medicine attendings and residents completed the stepwise, interactive module and then completed a survey to assess usability, face validity, content validity, and educational value.

Results

Participants rated the VR cricothyrotomy simulation as educationally valuable and effective for introducing procedural steps. However, usability fell below the acceptable threshold, with participants noting difficulties with hand control, depth perception, and feedback for correcting errors. The simulation was perceived as accurately representing all steps of a cricothyrotomy and providing clear instructional guidance. While patient tools were considered realistic, participants reported that the depth perception and control of the virtual hands were not fully realistic.

Conclusions

The VR simulation was designed to help novice learners build foundational knowledge before transitioning to physical training. Expert feedback suggests that the simulation offers educational value but could be enhanced through improvements to the user interface and haptics. As a low-risk method that does not require faculty guidance, this simulation has practical significance for early procedural training in medical education, particularly for high-acuity, low-frequency procedures.

## Introduction

Cricothyrotomy is an emergency procedure for airway access in a “can’t intubate, can’t ventilate” scenario. A study analyzing endotracheal intubations performed by emergency medicine residents over 58 months in the US and Canada reported a cricothyrotomy rate of 0.9%, highlighting its rarity in clinical practice [1]. Despite this infrequency, the Accreditation Council for Graduate Medical Education designates cricothyrotomy as a core procedure that must be completed during emergency medicine residency [2]. Because cricothyrotomy is both rare and typically performed in high-acuity situations, opportunities for direct clinical teaching are limited. Therefore, the quality of preclinical training for this lifesaving procedure is critically important.

Resident physicians must develop sufficient foundational knowledge of procedural steps before performing cricothyrotomy. Several high-fidelity simulators, such as cadaveric, porcine, and synthetic models, exist, but the limited time learners have with expert instructors necessitates maximizing simulation efficiency [3,4]. While traditional didactics provide a foundational understanding, they do not offer experiential procedural practice. Virtual reality (VR) training enhances learning by allowing repetitive rehearsal of procedure steps in an immersive, low-risk environment. This type of practice is especially valuable for high-acuity, low-frequency procedures. VR has been employed as a pre-operating room training tool, leading to improved performance [5-7].

In this study, we developed a VR cricothyrotomy simulation to provide learners with the opportunity to actively practice procedural steps before transitioning to physical simulation. The aim of this study was to evaluate the educational value, realism, and usability of this novel VR procedure simulation.

## Materials and methods

Participants

Fifteen emergency medicine attendings and residents participated by providing feedback either during an annual difficult airway lab or at their convenience from November to February 2025. Informed consent was obtained from all participants, and the protocol was approved by the University of Arizona IRB. 

Simulation development

The VR cricothyrotomy simulation (8Chili, Inc., Milpitas, CA, USA) was run on the HintVR simulation cloud using the HTC VIVE VR headset, both provided by 8Chili. The module was developed with input from a multidisciplinary team of healthcare professionals, including emergency medicine physicians, pulmonologists, pediatricians, surgeons, and nurse educators.

**Figure 1:**
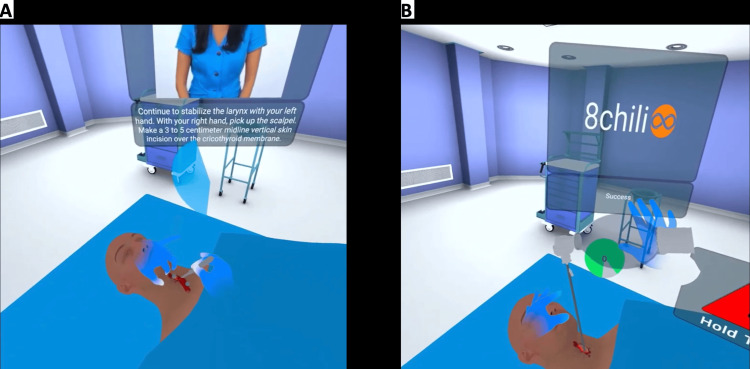
Learner view (A) Guidance provided while making the initial incision. (B) Successfully placed an endotracheal tube at the end of the simulation. Original image from the 8Chili interface (8Chili, Inc., Milpitas, CA, USA)

The VR simulation is a stepwise, interactive module that guides participants through each step of a bougie-assisted surgical cricothyrotomy. Participants must complete each step before advancing to the next, with instructional guidance provided throughout the procedure (Figure 1).

Data collection

Participants completed the VR cricothyrotomy simulation and filled out an anonymous survey. A simulation technologist was available during the simulation. The study was conducted at the Arizona Simulation Technology and Education Center (ASTEC) at the University of Arizona.

Demographic data collected included years since residency, prior experience with simulated cricothyrotomy, and clinical experience performing cricothyrotomy, including the number of procedures and success rate.

The survey was designed by the research team after reviewing relevant literature [8-13]. Four domains were assessed: usability, face validity, content validity, and educational value. Usability was measured with the 10-question System Usability Scale (SUS) [14]. Face validity, or the extent to which the simulation appears to accurately represent the procedure and relevant anatomy, was evaluated with nine questions. Content validity, or the extent to which the simulation includes all essential components, procedural steps, and anatomical features, was evaluated with six questions. Educational value was assessed with six questions on the effectiveness of the simulation as a teaching tool. All questions used a 5-point Likert scale (1 = strongly disagree, 5 = strongly agree), with a final free-text question for additional feedback (Appendix A).

To guide expert feedback, the survey included the VR simulation’s learning objectives, emphasizing skill development for novice learners: (1) understand the steps of a cricothyrotomy and the progression through each step; (2) demonstrate a cricothyrotomy procedure successfully establishing an airway; and (3) develop a simulation that can serve as an alternative or prerequisite to a cricothyrotomy procedural trainer (e.g., porcine or cadaver).

Data analysis

Survey data were entered into an Excel spreadsheet (Microsoft Corporation, Redmond, WA, USA). Responses were visualized using stacked bar graphs generated in Python (version 3.9.6) with Matplotlib and NumPy. Likert-scale responses are reported as the percentage of respondents selecting each option. Free-text responses were analyzed for common themes.

## Results

Participants

Fifteen emergency medicine attendings and residents participated, and their demographics are summarized in Table 1.

**Table 1:** Participant demographics

Participant demographics	Residents (n = 5)	Attendings (n = 10)
Mean years out of residency (y ± SD)	N/A	5.8 ± 4.4
Previous models of cricothyrotomy used (n)		
Porcine or other animal model	0	3
Simulation model	5	8
Cadaver	1	7
Experience performing a cricothyrotomy on a patient (n)	0	3
Mean number of cricothyrotomies performed on a patient (n ± SD)	0	1.3 ± 0.6
Cricothyrotomies successful in establishing an airway (%)	0	100

Usability

**Figure 2:**
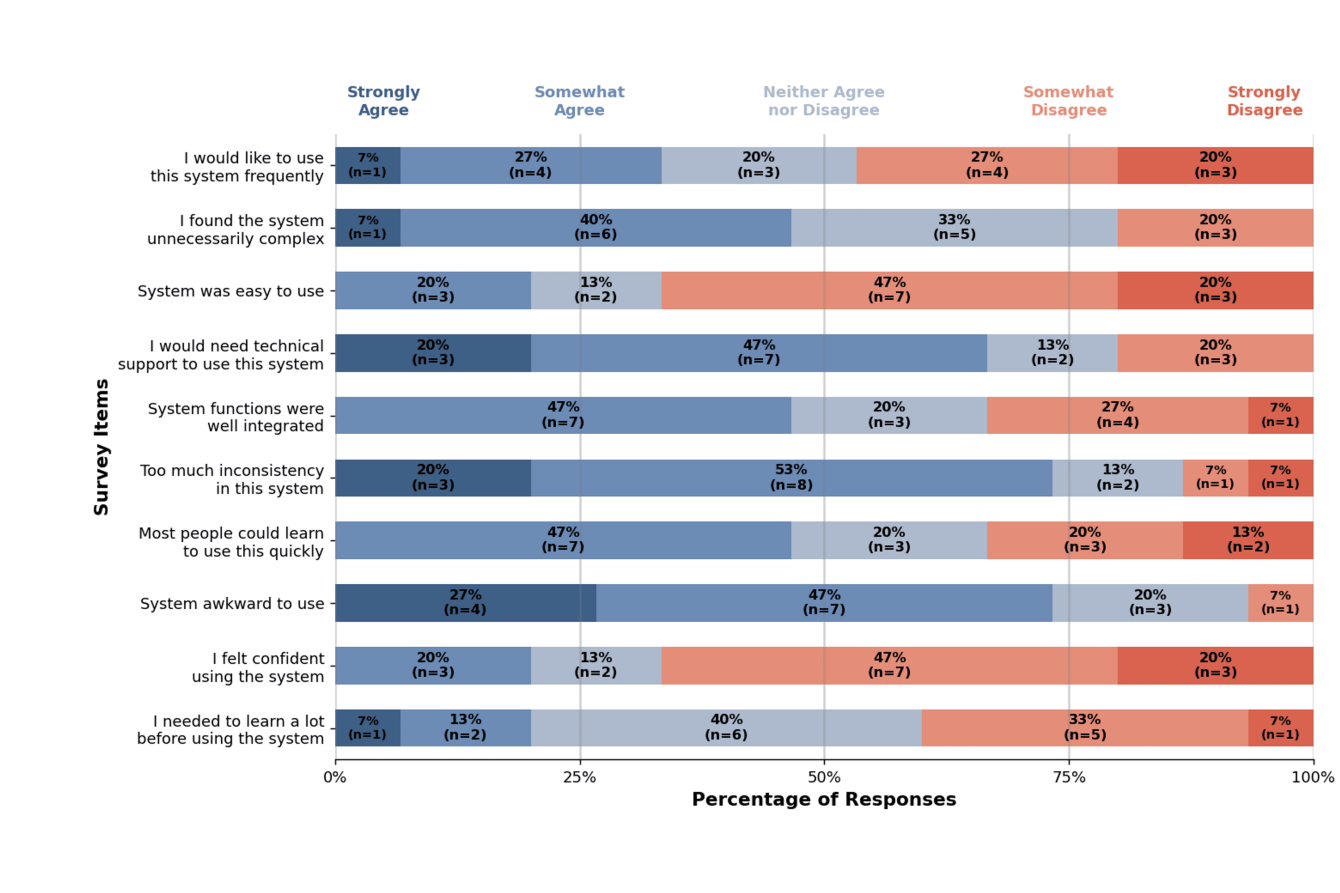
System usability results Percentage distribution of Likert scale responses for each usability survey item, with participant counts (n) shown for each response category. Total participants, n = 15.

The SUS included 10 questions, and responses are presented in Figure 2. Each participant’s responses were used to calculate an individual SUS score, with an average score of 40 [14]. This indicates that usability falls below the acceptable threshold, as scores below 50 are considered unacceptable [15].

Face validity

**Figure 3:**
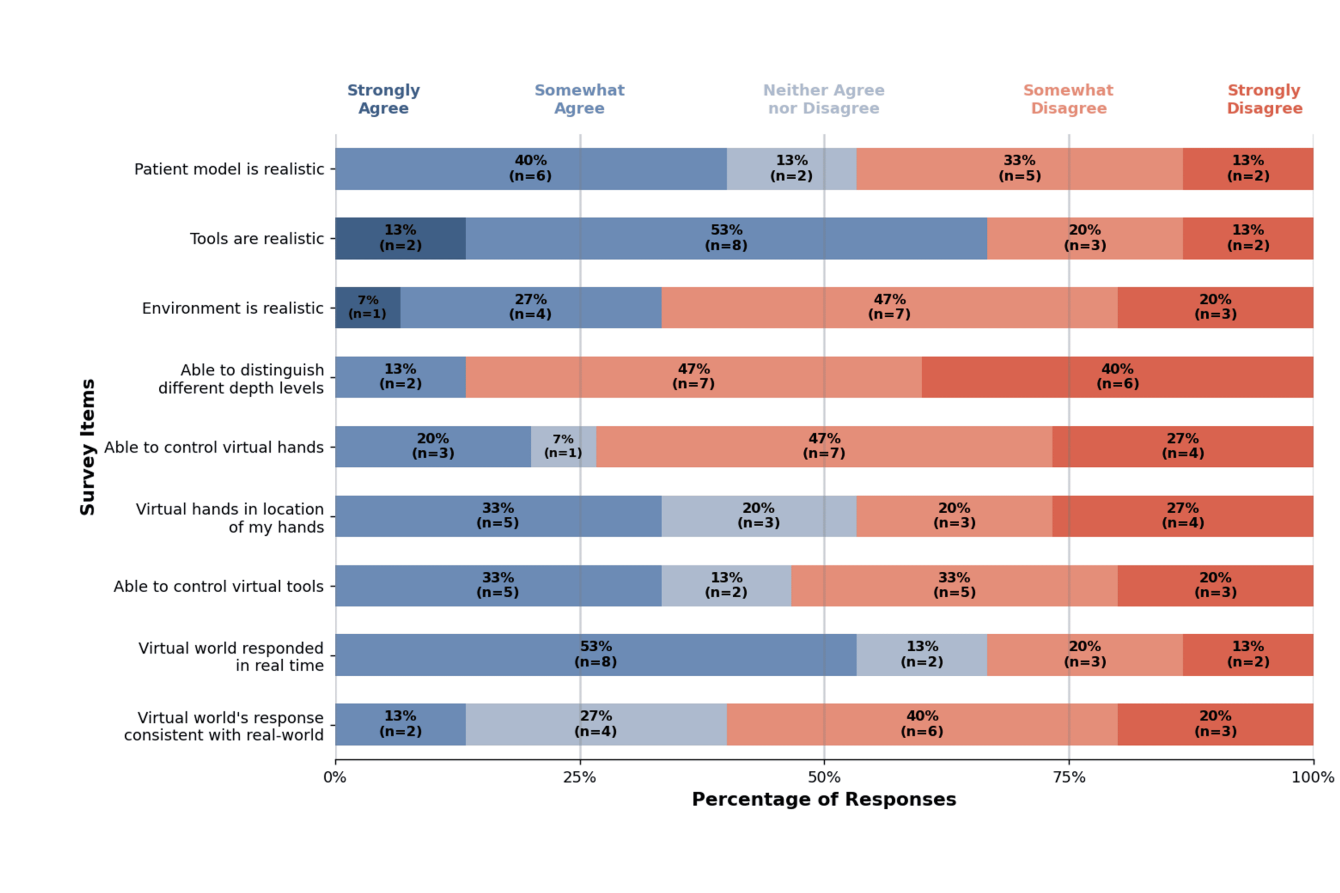
Face validity results Percentage distribution of Likert scale responses for each face validity survey item, with participant counts (n) shown for each response category. Total participants, n = 15.

The survey included nine questions assessing face validity, with responses summarized in Figure 3. Participants agreed that the tools and patient model were realistic. However, most participants selected “disagree” or “strongly disagree” for statements regarding depth perception and control of the virtual hands.

Content validity

**Figure 4:**
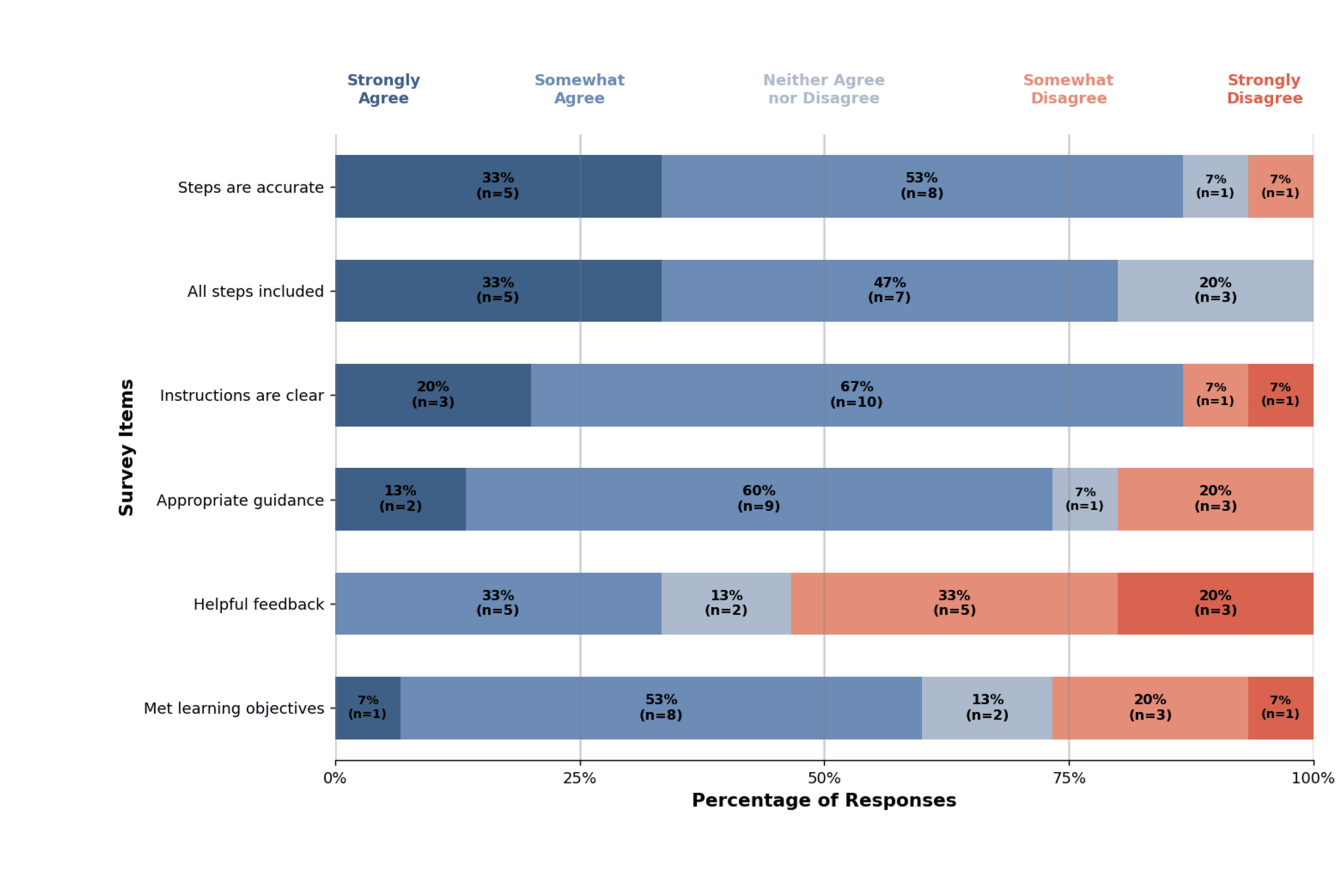
Content validity results Percentage distribution of Likert scale responses for each content validity survey item, with participant counts (n) shown for each response category. Total participants, n = 15.

Six questions assessed content validity, and responses are shown in Figure 4. Participants agreed that the simulation included all procedural steps accurately and that instructions were clear with appropriate guidance. However, only a few participants agreed that feedback was helpful in correcting mistakes.

Educational value

**Figure 5:**
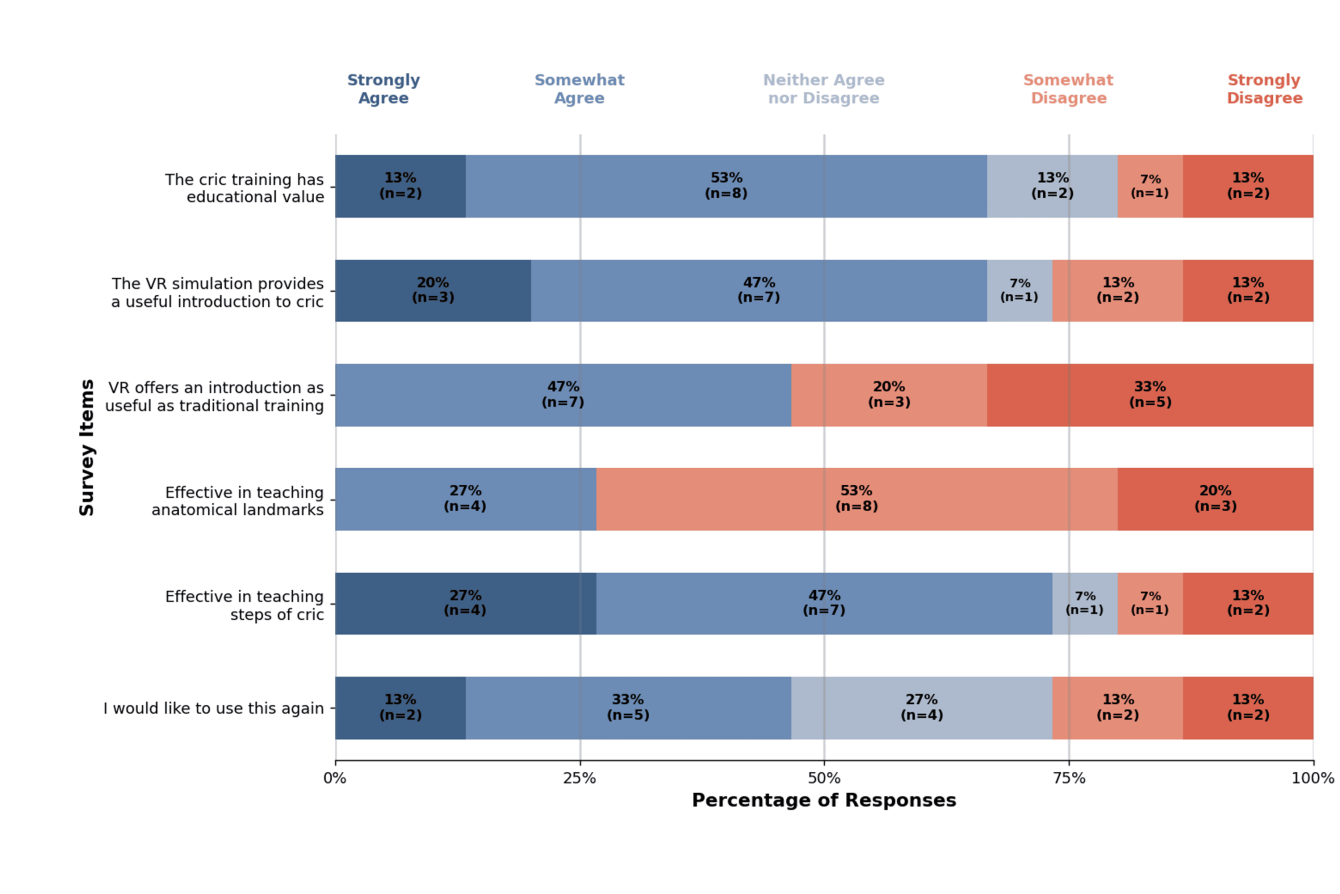
Educational value results Percentage distribution of Likert scale responses for each educational value survey item, with participant counts (n) shown for each response category. Total participants, n = 15.

Six survey questions addressed educational value, with results shown in Figure 5. The majority of participants agreed that the simulation was educational and provided a useful introduction to cricothyrotomy. Participants also judged the simulation effective for teaching procedural steps. However, participants disagreed that the simulation effectively taught anatomical landmarks for performing a cricothyrotomy.

Free response

Free-text responses were analyzed for qualitative themes and are summarized in Table 2. The most frequently reported concern was difficulty with hand orientation or manipulation (n = 9). Participants noted ergonomic discomfort during larynx palpation and challenges in grasping the cricothyroid. Six participants reported usability challenges, requiring technical assistance. Five respondents highlighted the lack of tactile feedback as a significant limitation. Despite these issues, four participants found the simulation valuable as an introductory tool for novices prior to practicing on physical models.

**Table 2:** Qualitative themes

Theme	Related comments
Lack of tactile feedback (n = 5)	“Cricothyrotomy is all about feel… does not replicate the necessary tactile input”; “Too difficult to use, no tactile response”; “Some sort of haptic feedback could be very helpful in gaining a sense of depth”
Visual clarity or display issues (n = 4)	“Dizzy, did not focus on my vision”; “Too blurry”; “Error messages would pop up in the periphery of my vision and disappear when I turned to look at them”
Difficulty using the system (n = 6)	“No direction on how to troubleshoot within the program - had to get the sim tech to help”; “Too difficult to use”; “If I hadn’t been told what to do, I may have given up”
Helpful for novices as an introduction to cric (n = 4)	“It is a great introduction tool before performing it in a simulation model or cadaveric model”; “May help identify anatomy and steps”; “The setup is very useful for learning the steps”
Hand orientation or manipulation issues (n = 9)	“The hand placement for palpation of the larynx was ergonomically uncomfortable”; “Difficult to do some of the steps, especially holding the cricothyroid”; “I couldn’t figure out how to grasp the larynx”

## Discussion

In this study, we developed a VR cricothyrotomy simulation to allow learners to actively practice procedural steps before transitioning to a physical model. The aim was to evaluate the usability, face validity, content validity, and educational value of this novel VR procedural simulation.

Usability

Participant feedback revealed an average SUS score of 40, below the acceptable threshold [15]. Free-response feedback highlighted themes consistent with the SUS, including issues with hand orientation, visual clarity, and system operation. Despite these usability challenges, survey responses indicated that most participants felt they could learn the system quickly with support. This aligns with prior research showing that successful VR training requires both learners and instructors to be familiar with VR prior to use [16]. Although a simulation technologist was available for troubleshooting, formal VR training was not provided, which may have contributed to the low usability scores. Future studies will include basic VR orientation before participation.

Several participants reported a blurry display, dizziness, or difficulty focusing. Providing orientation on headset adjustment may mitigate these issues and improve the learner experience. Nevertheless, cybersickness, a form of motion sickness associated with immersive environments, is well documented and varies between individuals. Strategies such as reducing visual latency and using lightweight headsets may lessen symptoms, but no solution is universally effective [17]. These limitations reinforce the role of the VR simulation as a preparatory tool rather than a replacement for physical, hands-on training. It provides valuable foundational exposure and should remain a supplement to in-person procedural education.

Face validity

Feedback on face validity was mixed. Most participants found the patient model, virtual tools, and real-time responsiveness realistic. However, the environment itself was not considered realistic, likely due to the stepwise, individual learner format rather than a team-based emergency scenario. The simulation’s primary goal was not to replicate the fast-paced environment of an emergent cricothyrotomy, but rather to build foundational procedural knowledge before engaging in advanced training. Although the VR environment may not fully replicate a high-stakes clinical scenario, it aligns with the objectives of understanding procedural steps, demonstrating successful airway establishment, and serving as a prerequisite to physical cricothyrotomy training.

While the virtual tools appeared realistic, over half of the participants reported difficulty controlling the virtual hands. This was the most common free-response theme, with nine participants noting hand orientation or manipulation issues. Future software updates will aim to improve hand tracking accuracy, enhance visual clarity, and address depth perception challenges, the most frequently cited face validity concern.

Content validity

Overall, participant feedback on content validity was generally positive, with most agreeing that the procedural steps were accurate, instructions were clear, and learning objectives were met. Interestingly, while over 70% of participants felt the simulation provided appropriate guidance, only 33% found the feedback helpful for correcting mistakes. This suggests that, although the step-by-step guidance aided task completion, the error feedback was insufficient for recognizing and addressing mistakes. Since this simulation targets novice learners, clear and actionable feedback on errors is essential. VR offers the opportunity for learners to receive real-time, individualized feedback, which has been shown to improve learning outcomes compared to VR without feedback [18,19]. Improving error-correction feedback will therefore be a priority in future versions of the simulation.

Although not evaluated in this study, the VR cricothyrotomy simulation includes a testing mode without stepwise guidance that provides an overall performance assessment. Expert feedback from this mode could be used to refine real-time guidance during procedural learning. As enrollment in nursing and medical programs continues to increase, accommodating in-person patient simulators or procedural task trainers with faculty supervision becomes increasingly challenging. VR simulations with integrated assessment and feedback capabilities offer a solution to these logistical and resource constraints, allowing learners to practice before high-stakes, summative assessments without requiring direct expert supervision.

Educational value

Overall, participants indicated that the VR cricothyrotomy simulation was valuable for teaching certain aspects of the procedure but identified areas for improvement to enhance educational effectiveness. The majority felt the training had educational value, particularly as an introduction to cricothyrotomy and for learning the procedural steps. This sentiment was reflected in free-response feedback, with four participants specifically noting its usefulness for novice learners.

The simulation was not considered effective for teaching anatomical landmarks critical to performing a cricothyrotomy. In free-response feedback, five participants highlighted the lack of tactile feedback as a limitation. This aligns with prior research showing that haptic feedback enhances procedural skill acquisition [20]. However, some studies suggest that the absence of haptic feedback may benefit novices by reducing cognitive load, allowing learners to focus on understanding and remembering the basic steps of the procedure [21]. While incorporating tactile feedback would improve realism, its absence does not appear to diminish the overall educational value of the simulation as an introductory training tool for cricothyrotomy.

Limitations

The VR cricothyrotomy simulation evaluated in this study was funded and developed by an industry partner. Although the research team conducted the study independently, this relationship should be considered when interpreting the findings. Additional limitations include the small number of participants with clinical experience performing a cricothyrotomy, as this procedure is extremely rare and most emergency medicine clinicians have never performed one outside of simulation. Future studies should aim to include more participants with real-world cricothyrotomy experience, including prehospital providers. Furthermore, research is needed to evaluate the extent to which skills acquired through the VR simulation transfer to physical simulation modalities and, ultimately, to real-world clinical procedures.

## Conclusions

We developed a VR cricothyrotomy simulation to help novice learners build foundational knowledge before transitioning to physical training. Expert feedback indicates that the VR simulation provides educational value, which could be further enhanced through improved learner orientation, user interface, and guidance for correcting mistakes. As a low-risk method that does not require faculty supervision, this VR simulation offers practical utility for early procedural training in high-acuity, low-frequency scenarios in medical education.

## Appendices

Appendix A

Demographic data

1. Please indicate how many years out of residency you are.

2. Please indicate your primary specialty:

3. What previous models of cricothyrotomy have you used? (select all that apply)

- Porcine or another animal model

- Simulation model

- Cadaver

4. In your clinical practice, have you performed a cricothyrotomy on a patient?

5. If you answered yes to the above question, please indicate how many times you have done this procedure on patients in your clinical practice.

6. What percentages of the cricothyrotomies performed were successful in establishing an airway?

Usability

Below are questions about usability of the virtual reality cricothyrotomy simulation. Your feedback is helpful in assessing the effectiveness of the virtual reality simulation in meeting these learning objectives:

- Understand the steps of a cricothyrotomy and the progression through each step.

- Demonstrate a cricothyrotomy procedure which is successful in establishing an airway.

- Develop a simulation which can be used as an alternative or prerequisite to a cricothyrotomy procedural trainer (ex. porcine or cadaver).

Please indicate how strongly you agree or disagree with each statement (Likert scale where 1 = strongly disagree, 5 = strongly agree).

1. I think that I would like to use this system frequently.

2. I found the system unnecessarily complex.

3. I thought the system was easy to use.

4. I think that I would need the support of a technical person to be able to use this system.

5. I found the various functions in the system were well integrated. 

6. I thought there was too much inconsistency in this system.

7. I would imagine that most people would learn to use this system very quickly.

8. I found the system very awkward to use.

9. I felt very confident using the system.

10. I needed to learn a lot of things before I could get going with this system.

Content validity

Below are questions about the content validity of the virtual reality cricothyrotomy simulation. Your feedback is helpful in assessing the effectiveness of the virtual reality simulation in meeting these learning  objectives:

- Understand the steps of a cricothyrotomy and the progression through each step.

- Demonstrate a cricothyrotomy procedure which is successful in establishing an airway.

- Develop a simulation which can be used as an alternative or prerequisite to a cricothyrotomy procedural trainer (ex., porcine or cadaver).

Please indicate how strongly you agree or disagree with each statement (Likert scale where 1 = strongly disagree, 5 = strongly agree).

1. The steps for performing a cricothyrotomy are accurate.

2. All the steps necessary for a cricothyrotomy are included.

3. The instructions are clear.

4. The program gave appropriate guidance. 

5. The program gave feedback that was helpful to correct mistakes.

6. The cricothyrotomy training met the stated learning objectives.

Face validity

Below are questions about the face validity of the virtual reality cricothyrotomy simulation. Your feedback is helpful in assessing the effectiveness of the virtual reality simulation in meeting these learning  objectives:

- Understand the steps of a cricothyrotomy and the progression through each step.

- Demonstrate a cricothyrotomy procedure which is successful in establishing an airway.

- Develop a simulation which can be used as an alternative or prerequisite to a cricothyrotomy procedural trainer (ex. porcine or cadaver).

Please indicate how strongly you agree or disagree with each statement (Likert scale where 1 = strongly disagree, 5 = strongly agree).

1. The patient model is realistic. 

2. The tools needed to perform a cricothyrotomy are realistic.

3. The environment is realistic in simulating the real world.

4. I was able to distinguish different depth levels while performing the procedure.

5. I felt I was in control of what the virtual hands did.

6. I felt the virtual hands were in the location of my hands.

7. I felt I was able to control the virtual tools.

8. The virtual world responded to my actions in real time.

9. The virtual world’s response to my actions was consistent with my real-world experience.

Educational value

Below are questions about the educational value of the virtual reality cricothyrotomy simulation. Your feedback is helpful in assessing the effectiveness of the virtual reality simulation in meeting these learning  objectives:

- Understand the steps of a cricothyrotomy and the progression through each step.

- Demonstrate a cricothyrotomy procedure which is successful in establishing an airway.

- Develop a simulation which can be used as an alternative or prerequisite to a cricothyrotomy procedural trainer (ex., porcine or cadaver).

Please indicate how strongly you agree or disagree with each statement (Likert scale where 1 = strongly disagree, 5 = strongly agree).

1. The cricothyrotomy training has educational value. 

2. The virtual reality simulation provides a useful introduction to cricothyrotomy.

3. Virtual reality offers an introduction to cricothyrotomy that is as useful as traditional hands-on training (porcine or synthetic model).

4. The virtual reality simulation was effective in teaching anatomical landmarks for performing a cricothyrotomy.

5. The virtual reality simulation was effective in teaching the steps of a cricothyrotomy.

6. I would like to use this again.

Please share any additional thoughts regarding aspects of the virtual reality cricothyrotomy simulation you found to be realistic or unrealistic.
